# An automatic pipeline for PET/MRI attenuation correction validation in the brain

**DOI:** 10.21203/rs.3.rs-2842317/v1

**Published:** 2023-05-17

**Authors:** Mahdjoub Hamdi, Chunwei Ying, Hongyu An, Richard Laforest

**Affiliations:** Washington University In St Louis: Washington University in St Louis; Washington University in St Louis School of Medicine Mallinckrodt Institute of Radiology; Washington University in St Louis School of Medicine Mallinckrodt Institute of Radiology; Washington University in St Louis School of Medicine Mallinckrodt Institute of Radiology

**Keywords:** Quantitative brain PET, PET attenuation correction, PET/MRI, PET/CT, FreeSurfer brain atlas, Virtual synthetic PET imaging

## Abstract

**Purpose:**

PET/MRI quantitative accuracy for neurological applications is challenging due to accuracy of the PET attenuation correction. In this work, we proposed and evaluated an automatic pipeline for assessing the quantitative accuracy of four different MRI = based attenuation correction (PET MRAC) approaches.

**Methods:**

The proposed pipeline consists of a synthetic lesion insertion tool and the FreeSurfer neuroimaging analysis framework. The synthetic lesion insertion tool is used to insert simulated spherical, and brain regions of interest (ROI) into the PET projection space and reconstructed with four different PET MRAC techniques, while FreeSurfer is used to generate brain ROIs from T1 weighted MRI image. Using a cohort of 11 patients’ brain PET dataset, the quantitative accuracy of four MRAC(s), which are: DIXON AC, DIXONbone AC, UTE AC, and Deep learning trained with DIXON AC, named DL-DIXON AC, were compared to the PET-based CT attenuation correction (PET CTAC). MRAC to CTAC activity bias in spherical lesions and brain ROIs were reconstructed with and without background activity and compared to the original PET images.

**Results:**

The proposed pipeline provides accurate and consistent results for inserted spherical lesions and brain ROIs inserted with and without considering the background activity and following the same MRAC to CTAC pattern as the original brain PET images. As expected, the DIXON AC showed the highest bias; the second was for the UTE, then the DIXONBone, and the DL-DIXON with the lowest bias. For simulated ROIs inserted in the background activity, DIXON showed a −4.65% MRAC to CTAC bias, 0.06% for the DIXONbone, −1.70% for the UTE, and - 0.23% for the DL-DIXON. For lesion ROIs inserted without background activity, DIXON showed a −5.21%, −1% for the DIXONbone, −2.55% for the UTE, and - 0.52 for the DL-DIXON. For MRAC to CTAC bias calculated using the same 16 FreeSurfer brain ROIs in the original brain PET reconstructed images, a 6.87% was observed for the DIXON, −1.83% for DIXON bone, −3.01% for the UTE, and - 0.17% for the DL-DIXON.

**Conclusion:**

The proposed pipeline provides accurate and consistent results for synthetic spherical lesions and brain ROIs inserted with and without considering the background activity; hence a new attenuation correction approach can be evaluated without using measured PET emission data.

## 1. Introduction

PET attenuation correction (AC) is crucial for accurate PET quantification [1]. Shortly after its introduction, the hybrid PET/CT imaging system, CT AC (CTAC), has since been considered the gold standard for PET attenuation correction [2]. Hybrid PET/MRI gained interest due to its intrinsic high soft-tissue contrast, especially for neurological and oncology applications [3]. Hybrid PET/MRI imaging systems were introduced a decade ago [4]. However, PET/MRI is still mainly used in the research arena due to cost and lack of unique applications that would require simultaneity of PET and MR acquisition. In addition, PET attenuation correction using MRI information is not straightforward. MRI images provide information about tissue proton density and relaxation times but not tissue electron densities. Thus, body tissues with low proton densities or with very short relaxation time are not seen by the MRI system. The most important consequence is the absence of bone tissues in MRI AC (MRAC) images affects the quantitative accuracy of the PET reconstructed images [5]. Research literature about MRAC for PET can be sorted into three categories [6]. The first category is based on tissue segmentation of MRI images, for instance, the DIXON to identify water-like and fat-like tissues and UTE MRI imaging sequences [7] to attempt to localize the bones. A second category is a templates-atlas-based approach, such as the superimposition of a bone template on a Dixon attenuation map [8]. And the third category is based on deep learning techniques [9]. Several published studies reported that the MRAC issue was solved for healthy subjects with normal anatomies, especially in the brain, and reported MRAC approaches with similar performance to CTAC [10]–[12]. However, this is not the case for subjects with skull and brain abnormalities, where it is difficult to assess the performances of the template-atlas and deep learning MRAC approaches due to the lack of availability of these patient datasets with particular abnormalities. For instance, implementing these approaches, the atlas-template and deep learning-based MRAC approaches in a clinical routine need further evaluation and continuous improvement using a large patient cohort with diverse anatomies and pathologies [13].

Virtual Synthetic PET imaging has gained attention as a tool for overcoming the issue of diversified patient datasets. There are three types of virtual synthetic PET imaging techniques, analytical, probabilistic, and deep learning [14]. Analytical and probabilistic methods have the advantage of knowing the ground truth over deep learning techniques. Ground truth enables PET-quantitative accuracy evaluation where many sources of biases are merged in the final reconstructed images. Analytical synthetic PET imaging simulation provides enough realistic acquisition, and PET images are obtained in a few minutes on a standard workstation, compared to Monte Carlo Simulations, where one realistic PET simulation takes days on parallel computing infrastructure or GPUs [15].

This paper presents an analytical brain PET synthetic imaging pipeline-specific evaluation of attenuation correction methodologies in neurological PET/MRI studies. The pipeline uses a previously developed synthetic lesion insertion tool [16] and the FreeSurfer framework [17]. The pipeline was used to evaluate several common MRAC approaches on PET quantitative accuracy relative to the CTAC approach as a reference for brain PET quantitative accuracy.

## 2. Method

### a. Patient data

Eleven patient brain PET datasets that were part of a clinical research study at our institution were used for this study. The Institutional Review Board approved the clinical research study protocol, and the patients provided written consent. Single time tri-modality brain PET/MRI/CT images were acquired for each patient. Patient brain PET emission data and brain MRI images were obtained from a Biograph mMR PET/MRI system (Siemens Healthcare). Head CT images were obtained with the clinical Biograph True Point 40 PET/CT system (Siemens Healthcare). The quantitative accuracy of the brain PET images was evaluated using four MRAC approaches and compared to CTAC as a reference. The two-point DIXON MRI sequence (DIXON) [7] segmenting head tissue as fat and water like only, the same two-point DIXON but including a skull model (DIXONbone) [18], the ultra-short echo-time MRI sequence (UTE) [19] that extract bone information from short relaxation time of protons in bone, and a DIXON trained deep learning network generated pseudo-CT map (DL-DIXON) developed by our group [20]. Three MRAC approaches, DIXON, DIXONbone, and UTE, are available on the mMR PET/MRI system. T1 MPRAGE MRI brain images from each patient were used as input to the neuroimaging data analysis framework FreeSurfer and provided a patient-specific brain atlas representing 15 regions. Brain ROIs were used to define the shape and location of lesions in the synthetic lesion insertion tool.

### b. PET imaging

Patients were injected with an F-18-based amyloid binding radio-ligand (Florbetapir). Data was acquired 50 minutes post-injection for 30 minutes scan duration, except one patient was measured 63 minutes post-injection and scanned for 68 minutes, using the mMR PET/MRI system. List-mode files were acquired and re-binned to sinograms using the Siemens research reconstruction software e7tools (Siemens healthcare). PET images were reconstructed using a 3D OSEM algorithm at 3 iterations, 24 subsets, and a 4 mm post-reconstruction Gaussian smoothing kernel [21]. The PET reconstructed image sizes are 344 x 344 x 127 voxels at 2.08 x 2.08 x 2.03 mm^3^ each.

### c. CT imaging

A low dose brain CT images were acquired using the CT subsystem of the Biograph TruePoint 40 PET/CT scanner at 120 kVp, 25 mAs exposure. Images were reconstructed using the filtered back-projection algorithm with H19f. The dimension of the brain CT images is 512x512x70 voxels at 0.59 x0.59x2 mm3 per voxel.

### d. MRI imaging

Three brain MRI images were acquired using the mMR PET/MRI system using vendor-provided sequences, the standard two-point Dixon 3-dimensional Volume Interpolated Breath-hold Examination (VIBE), the high-resolution two-point Dixon CAPI, UTE, and the Magnetization-prepared Rapid Acquisition Gradient Echo (MPRAGE).

MRI T1-weighted brain images were acquired using a 3D MPRAGE sequence with the following imaging parameters: TE/TR = 2.95/2300 ms, TI = 900 ms, number of partitions = 176, matrix size = 240 × 256 × 176, voxel size = 1.05 × 1.05 × 1.2 mm3, acquisition time = 5 min 11 s. The T1-weighted image was used as an input to FreeSurfer to generate the patient specific brain atlas.

### e. Attenuation maps

#### DIXON

The DIXON attenuation map was acquired using a vendor-provided two-point Dixon VIBE MRI sequence with a 10-degrees flip angle. At repetition time (TR), 3.6 ms, there are two echo-time TE readouts, in-phase, 2.46 ms, and out-phase, 1.23 ms, from which fat and water dominant images are generated. The acquisition time was 19 sec. Four classes of tissues are generated for whole-body PET/MRI applications: air, fat, and soft tissues, to which fixed 511 keV photons attenuation coefficient were assigned. In this study, the whole brain and the skull are considered uniform soft tissues (water). The dimensions of the DIXON images are 192 × 126 × 128 voxels, and the voxel size is 2.6 × 2.6 × 3.12 mm^3^.

#### DIXONbone

The DIXONbone images were generated based on the high-resolution DIXON CAPI images (TE1/TE2/TR = 1.28/2.51/4.14 ms, FA = 10 degrees, dimensions = 384x204x132 voxels, and voxel size = 1.30x1.30x2.02 mm, acquisition time = 39 sec) Skull bones linear attenuation coefficient (LAC) replaced the soft tissues LAC in the high-resolution DIXON images. The skull bones were generated using a vendor-provided model-based bone prototype segmentation algorithm (Siemens AG, Erlangen, Germany). The first step is to generate a bone model from pre-aligned MRI images and bone masks containing continuous bone LAC at 511 keV photons. In the second step, the patient DIXON image is registered to the generated MRI model, then bone masks are registered to the patient DIXON image, segmented in the bone tissues, and brought back to the original DIXOM image space using the same transformations [18], [22].

#### UTE

The UTE images were generated using a vendor-provided MRI imaging sequence with a 10-degree flip angle, 4.64 ms repetition time (TR), and 0.07 ms and 2.46 ms echo time, which results in simultaneous generation of cranial bones and the brain tissues. The acquisition time is 144 seconds. The resulted images were segmented into two compartments, soft tissues for the whole brain and bones-tissues for the cranial bones. The size of the raw UTE images consists of 192x192x192 voxels, 1.56x1.56x1.56 mm3 per voxel.

#### DL-DIXON

Synthetic pseudoCT attenuation maps were generated using a deep-learning technique. A network that combines the 3D residual and UNet architectures (ResUNet) was used. Pseudo-CT images were generated from the standard in- and opp-phase DXION images. More details about the DL-DIXON attenuation maps generation methodology, network architecture, training and testing datasets were published in previous work in [23].

### f. Attenuation maps preprocessing

The four MRI-derived attenuation maps and CT images obtained directly from the Biograph mMR, and True Point 40 PET/CT systems were resampled using nearest neighbor interpolation onto the default attenuation map gridded on 344 x 344 x 127 voxels, a 2.086 x 2.086 x 2.031 mm3. The DIXON, DIXONbone, and DL-DIXON MRAC were registered to the UTE attenuation map using a 12-parameter affine registration with the FMRIB Linear Image Registration Tool (FLIRT) in the FSL toolbox [24]. The CT and pseudo-CT numbers were converted to 511 KeV linear attenuation coefficients by piecewise linear scaling [25].

The CT and pseudo-CT Hounsfield unit were converted to 511 KeV linear attenuation coefficients by piecewise linear scaling [25]. The CT attenuation maps were aligned to DIXON, DIXONbone, UTE and DL-DIXON attenuation maps using a 12-parameter affine registration with FLIRT. All MR and CT attenuation maps were then wrapped to default attenuation map space (344 x 344 x 127 voxels, a 2.086 x 2.086 x 2.031 mm^3^) using vendor-provided JSRecon software.

### e. Pipeline description

**Figure 1:**
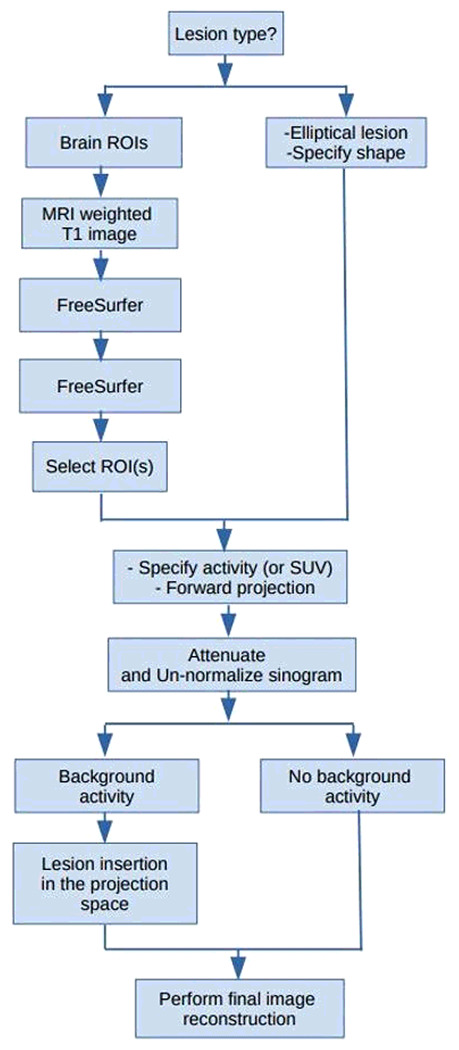
Pipeline for the evaluation of different PET attenuation correction approaches. The synthetic lesion ROIs insertion tool and FreeSurfer.

The pipeline consists of exploiting a synthetic lesion insertion tool validated for the Siemens mMR and FreeSurfer, a neuroimaging data analysis framework used for brain segmentation purposes. The mMR synthetic lesion insertion tool was validated using contrast recovery coefficients in six spheres NEMA IEC phantom and preliminarily evaluated for MRAC to CTAC activity bias in an elliptical lesion inserted in two anatomical regions, the brain and the pelvis, and published in previous work [16]. A flowchart of the pipeline is presented in Fig. 1. First, the T1 weighted MRI images are input to FreeSurfer to generate a patient-specific brain atlas. Then, for each patient, one or multiple brain ROIs are used as input to the synthetic lesion insertion tool. ROI activity or SUV can be defined as a uniform value or lesions to background ratio (LBR). In the latter, the original PET image needs to be provided with the activity in the given brain ROI, multiplied by the LBR, and smoothed with scanner point spread function (PSF). Then the lesion ROI(s) is forward projected to sinogram space, attenuated and normalized. Scatter events and Poisson noise is then added. The lesion sinogram is either added to or replaced the sinogram counts in the patient PET projection space. Finally, a PET image is reconstructed (Fig. 1) using standard 3D-OSEM [21]. More detail about the lesion insertion tool is presented in [16].

### f. Brain region of interests

**Figure 2:**
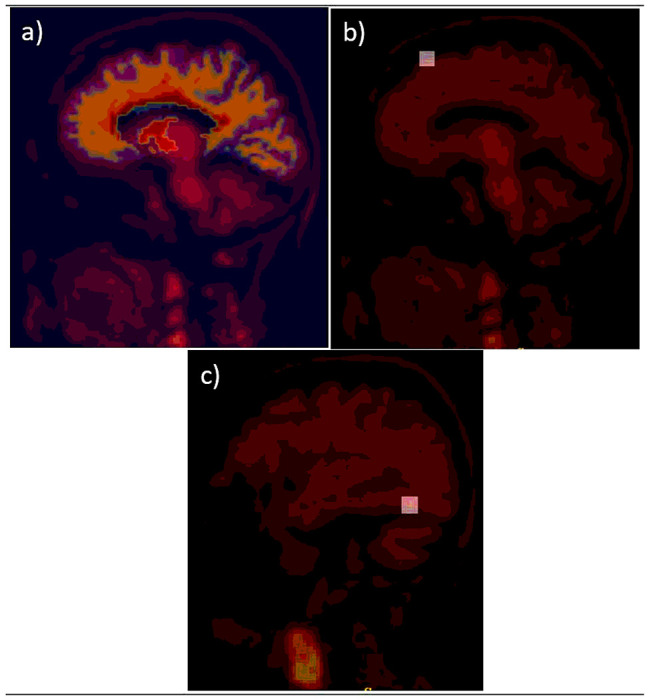
Example of inserted FreeSurfer brain atlas generated using T1 MRI brain image (a) and spherical lesions (b) and (c). The inserted lesions and atlas are superposed on the PET image.

In a typical comparison of PET/MRI to PET/CT for attenuation correction evaluation, the PET emission data is reconstructed with two different attenuation maps: a specific MRAC and a CTAC. For regional brain uptake analysis, brain ROIs are delineated using manual or automatic approaches to calculate the uptake deviation from MRAC PET reconstruction relative to CTAC PET reconstruction. In the case of automatic ROIs generation, for instance, a brain atlas generated from a FreeSurfer T1 weighted MRI images with 256x256x256 voxels at ~ 1mm3/voxel needs to be in the same space as the final reconstructed PET images. The FreeSurfer brain atlases were aligned to the PET using rigid registration with FSL’s FLIRT Figure 2a presents a 2D slice of a brain atlas superimposed on its corresponding 2D brain PET image.

### g. Pipeline evaluation

A spherical 4 mm radius lesion was inserted at two positions in two brain ROIs, in the superior frontal cortex and the fusiform gyrus, to keep the same position across brain PET patients’ datasets as much as possible. A larger difference in brain uptake quantitative accuracy assessment is most sensitive to a location near the bones. Inserted lesions were reconstructed with and without considering the brain PET background (or projections) and reconstructed using the four MRAC maps, DIXON, DIXONbone, UTE, DL-DIXON, and the CTAC map. Normal original brain PET images were also reconstructed in the same way. MRAC to CTAC bias in lesions inserted with and without background can be compared to MRAC to CTAC bias in the original PET images. Figure 2b and c shows an example of the two spherical lesions inserted in the two different brain ROIs.

### h. Data analysis

MRAC to CTAC bias was calculated in the inserted spherical lesions and FreeSurfer brain atlas ROIs with and without background and in the normal original PET images using Eq. 1. Box plots of the average MRAC to CTAC bias were displayed for the entire patient cohort.


$$bias={\left(PET_{MRAC}-PET_{CTAC}\right)/PET}_{CTAC}$$

## 3. Results

Brain lesions ROIs activity bias in PET reconstructed images from four MRI-derived attenuation correction maps were evaluated against a CT attenuation map using synthetically inserted spherical lesions, and subject-specific brain ROIs were extracted using FreeSurfer. In addition, MRAC to CTAC activity bias was calculated in lesion and ROIs inserted with and without patient background and in the original brain PET images (no synthetic lesion or ROIs insertion). Results are presented for 16 brain ROIs forming three major brain regions, the frontal cortex and the parietal and temporal cortex.

### a. Pipeline evaluation

**Figure 3:**
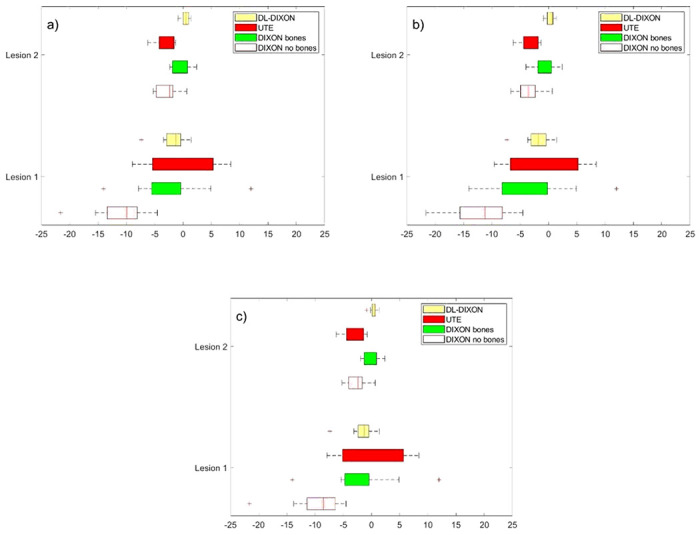
MR to CT-based PET attenuation correction bias in two synthetic spherical lesions inserted in different brains ROIs. a) Lesions are reconstructed by adding the lesion and the patient emission sinograms. b) Inserted lesions were reconstructed using only lesions emission sinogram. c) The corresponding ROIs in the original PET images.

Figure 3 presents a boxplot of the MRAC to CTAC lesion’s activity bias for the 11 patient datasets. Figure 3a shows the bias in lesions reconstructed with background activity. Figure 3b shows MRAC to CTAC lesion activity bias calculated in lesions inserted without considering the patients’ PET sinogram; hence, only the inserted spherical lesions are reconstructed in the final image. Figure 3c shows the MRAC to CTAC bias lesions like spherical ROIs in the original PET images. MRAC to CTAC activity bias in the original PET images shows similar behavior to the inserted lesions for the four MRAC approaches (Fig. 3a, Fig. 3b, and Fig. 2c). For lesions inserted in the superior frontal cortex, at the vicinity of the skull, the DIXON AC showed the largest underestimation of activity with a median of −3.26% [−6.08%, −2.14%] and an interquartile range for lesion inserted in the activity background. Lesion inserted without the activity background showed a median of −3.80% [−6.10%, 2.16%]. The UTE showed an enhanced activity estimation compared to the DIXON AC, −0.57% median, but a larger fluctuation of the average of MRAC to CTAC activity bias with an interquartile range (IQR) of 8.04% (−1.99–6.05%) for lesion inserted in the background activity and 0.88% in median and 7.79% IQR (−0.86–6.92%) for lesion inserted without considering the background activity. The DIXONbone showed enhanced performances than the UTE with − 0.29% median and less fluctuation with an IQR of 4.68% (−2.92–1.75%) for lesions inserted in the background activity. Lesion inserted without the background activity showed a median of −0.16% and an IQR of 4.68% (−2.56–2.12%). The DL-DIXON showed the best performance, with an MRAC to CTAC activity bias of −0.11% in median and 1.5% IQR (−0.38–1.12%) for lesions inserted in the background activity and − 0.35% in median and 1.13% IQR (−0.13–1%) for lesions inserted without the background activity.

In lesion inserted in the fusiform gyrus, which is part of the temporal lobe and occipital lobe in Brodmann area, not in the vicinity of the skull, the four attenuation maps showed an MRAC to CTAC activity bias with the same order as for lesion inserted in the superior frontal cortex, except for the UTE MRAC, for both lesions inserted with or without considering the background activity. The UTE in lesion two showed a lower MRAC to CTAC bias and interpatient variability, with a −3.70% median and 2.78% IQR (−4.78% to −2%) for lesion inserted in the background activity. Similarly, for lesions inserted without considering the background, −3.88% median and 3.66% IQR (−5.24% to −1.58%). The DL-DIXON showed the lowest lesion activity bias with a median value of −0.67% and 1.34% IQR (−1.15–0.18%) for lesions inserted on the background the medium and IWR values were − 0.77% and 1.34% (−1.17–0.17%). DIXONbone showed similar performances to the DL-DIXON with a median value within - 1% and an IQR within 2% for lesions inserted with and without considering the background activity.

The MRAC to CTAC bias in the inserted lesions, shown in Fig. 3a. and Fig. 3b., show good agreement with MRAC to CTAC lesion bias calculated in their corresponding original reconstructed PET brain image (Fig. 3c).

### b. Regional Brain ROIs

**Figure 4:**
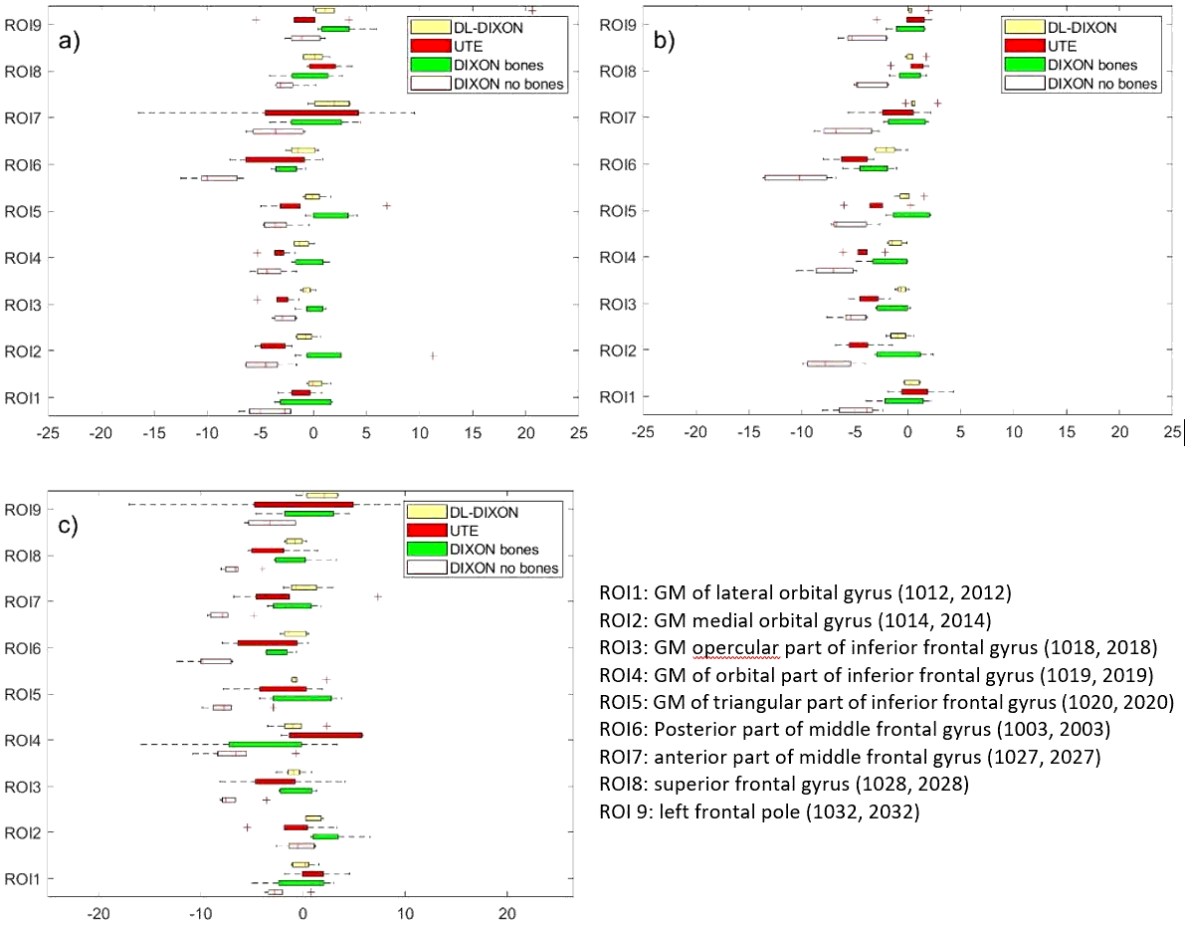
MRAC to CTAC based PET attenuation correction bias a FreeSurfer extracted T1 ROIs in the prefrontal cortex. a) ROIs are reconstructed by adding the ROI and the patient emission sinograms. b) Inserted ROIs were reconstructed using only lesions emission sinogram.

Figure 4 presents MRAC to CTAC activity bias in nine lesion ROIs in the prefrontal cortex. MRAC to CTAC activity bias in lesion ROIs inserted with and without considering the background activity is presented in Fig. 4a and Fig. 4b. Figure 4c shows activity bias in the original reconstructed PET images using the same ROIs.

Lesions ROIs inserted in the prefrontal cortex with and without considering the background activity showed similar behavior to lesion ROIs in the original brain PET reconstructed images. However, a slightly higher fluctuation, activity bias range, was observed in the original lesion ROIs due to their lower statistics than the inserted synthetic lesions ROIs. For lesions ROIs in the prefrontal cortex, inserted on the background activity, the DIXON AC showed the largest underestimation of activity, with a median ranging from 0.48% in the left frontal pole to −7.20% in the caudal middle frontal gyrus and present the lowest and highest MRAC to CTAC activity biases, respectively (Fig. 4). The UTE AC showed an enhancement in activity estimation relative to the DIXON, with an MRAC to CTAC activity bias with a median ranging from 0.57–3.7% for both lesions inserted with or without the background activity. However, UTE has higher interpatient variability, IQR (Fig. 4a.). The DIXONbone performed better than the UTE, with a median activity deviation ranging from 0.01–1.99%. The DIXON-DL presented the best performance across all MRAC approaches, with the lowest MRAC to CTAC activity bias with a medium ranging from 0.11–0.76%. A similar pattern was observed using MRAC to CTAC activity bias in lesion ROIs inserted without considering the background activity and lesion ROIs in the original reconstructed images (Fig. 4b.). MRAC to CTAC lesion ROIs bias shows good agreement with MRAC to CTAC bias calculated with the same ROIs in the original brain PET images. However, the original brain PET images have more interpatient variability (Fig. 4c.).

**Figure 5:**
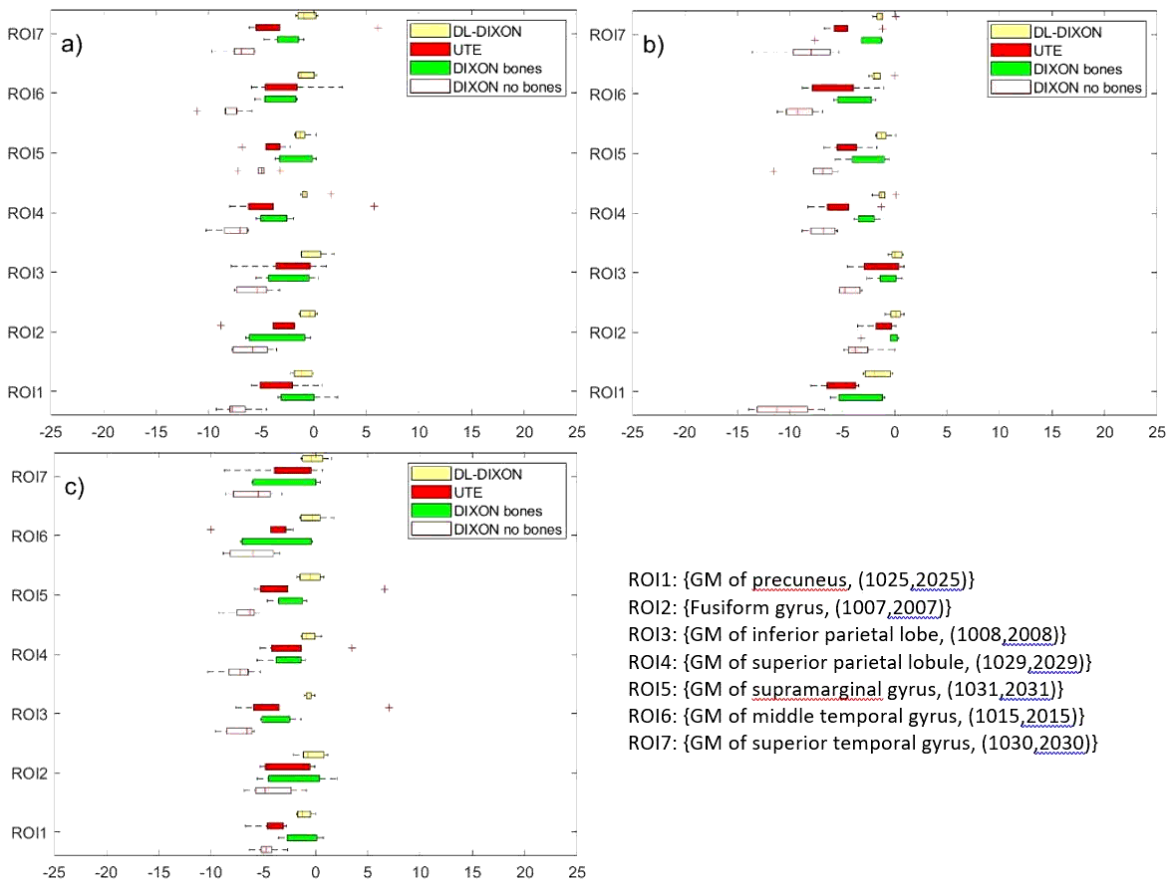
MR to CT based PET attenuation correction bias a FreeSurfer extracted T1 ROIs in the parietal and temporal cortex. a) ROIs are reconstructed by adding the ROI and the patient emission sinograms. b) Inserted ROIs were reconstructed using only lesions emission sinogram.

Figure 5 depicts MRAC to CTAC bias in brain ROIs in the parietal and temporal cortexes. Lesion ROIs inserted with and without the background activity show similar MRAC to CTAC bias for the four MRAC approaches. First, DIXON AC showed the largest underestimation of the activity with a median value ranging from − 4.82% to −7.31% in lesion ROIs inserted in the background activity and − 3.3% to −8.31% for lesion ROIs inserted without background activity. The UTE AC showed the second largest bias with a median value activity bias ranging from − 2.03% to −4.42% for lesion ROIs inserted in the background activity and − 0.04–5.32% for lesion ROIs inserted without the background activity. Then the DIXONbone showed an enhancement compared to the DIXON and UTE with a median value ranging from 0.01% to −2.56% for lesion ROI inserted in the background activity and a −0.14% to −2.58% in lesion ROIs inserted without background activity. Finally, the DL-DIXON presented the best performances across the four MRAC approaches with a median activity bias ranging from − 0.01–0.76% for lesion ROIs inserted in the activity background and 0.01% to −1% in lesion ROIs inserted without the background.

## 4. Discussion

Our developed pipeline analytically generated realistic 3D PET brain ROIs and lesions. The tool was used to evaluate the quantitative accuracy of different MRI-based PET attenuation correction approaches to CT-based attenuation correction and showed a very good agreement with the original measured patient PET data and will enable mimicking clinically relevant diseases in the brain studied by PET. Different brain anatomical regions in the gray matter were investigated, including the prefrontal, parietal, temporal cortexes, and the fusiform gyrus. Three types of results were presented in this study, 1- lesions inserted with the PET projection space, 2-lesions inserted without the patient PET emission data, and 3- the original PET image. Lesions inserted without the PET emission data showed comparable behavior to lesions inserted with PET emission data and in the original reconstructed PET images. This paper’s finding suggests using the synthetic lesion insertion tool to evaluate the quantitative accuracy without the need for PET emission data.

Available clinical PET emission or transmission data can also be diversified and augmented due to the lack of pathological data needed to evaluate the quantitative accuracy, image reconstruction algorithms, hyper-parameters, and data processing, like segmentation.

Beatrice et al. [26] provided a synthetic lesion insertion tool for evaluating PET automatic segmentation approaches. The lesion is inserted into the projection space obtained by forward projection of an already reconstructed PET image, using MATLAB’s Radon transform, which makes the lesion insertion tool independent of the scanner geometry. The downfall of this approach is that the inserted lesion should be at least 3 cm away from the phantom edge, and the results should be calibrated for a specific scanner. This cannot be used for the assessment of the quantitative accuracy of the PET/MRI image near the cranial bones, for instance. In our case, the vendor forward projector was used to preserve the same lesion projection geometry to be added (or replaced) to the patient’s sinogram.

Other studies used Monte Carlo simulation codes to generate a realistic PET lesions database that mimics clinical tumor heterogeneity from previously reconstructed images [27]. However, generating such realistic datasets needs an extensive validation of the Monte Carlo Model of the scanner, i.e., the scanner detector response. In our case, by using a matching forward projector specific for the scanner, i.e., Biograph mMR, analytically inserted lesion ROIs has a good agreement with the scanner data in less than five minutes simulation time on a standard workstation with Intel(R) Xeon(R) CPU X5650 at 2.67GHz (2 processors).

As expected, among the three vendor-provided attenuation approaches available for the Siemens Biograph mMR users, the DIXONbone outperformed the standard DIXON without bones and the UTE. The UTE came in second place after the DIXONbone. The DIXON without including bones has the lowest quantitative accuracy among all. Our findings are consistent with the published literature [8].

The median MRAC to CTAC activity bias of the 16 inserted lesion ROIs for the 11 patients, whether with or without the background activity and in the original PET patient brain images, followed the same pattern. For lesion ROIs inserted in the background activity, DIXON AC showed a −4.65% (IQR − 5.39% to −1%) MRAC to CTAC bias, 0.06% (IQR − 2.08–1.39%) for the DIXONbone, −1.70% (−2.74% to −0.69%) for the UTE, and − 0.23% (−0.72–0.33%) for the DL-DIXON. For lesion ROIs inserted without background activity, DIXON showed a −5.21% (IQR − 7.74% to −3.03%), −1% (IQR − 2.88% to −0.03%) for the DIXONbone, −2.55% (IQR − 3.56% to −1.50%) for the UTE, and - 0.52 (−0.79–0.19%) for the DL-DIXON. For MRAC to CTAC bias calculated using the same 16 FreeSurfer brain ROIs in the original brain PET reconstructed images, a 6.87% (IQR − 8.13 to −4.10%) was observed for the DIXON, −1.83% (IQR – 3.79% to −0.32%) for DIXON bone, −3.01% (IQR − 4.08% to −1.50%) for the UTE, and − 0.17% (−0.79–0.47%) for the DL-DIXON. These results suggest that a new attenuation correction approach can be evaluated without using measured PET emission data. The deep learning-based attenuation correction approach showed the best quantitative accuracy among the other approaches evaluated.

The synthetic lesion insertion tool has the potential to accelerate the development, evaluation, and translation of new attenuation correction approaches to commercial solutions. In addition, the existing approach can also be enhanced and evaluated right away using the synthetic lesion insertion tool.

Lesions ROIs are attenuated using the real attenuation map, which limits quantitative bias compared to other studies that assume water to attenuate the inserted lesions. However, the correctness of the brain atlas needs to be accurately registered to the attenuation map.

One interesting avenue is studying the effect of anatomical change on the quantitative deviation accuracy of PET images. The proposed pipeline can be used to simulate clinically relevant neurological diseases from healthy patient data. This data will be used to diversify a deep-learning database. The lesion insertion tool will be available through an online interface where the user can load the attenuation map or the PET emission projection data.

In the original PET images, larger ROIs have more fluctuation in MRAC to CTAC bias compared to smaller ROIs. One reason might be the lower statistics in the lesions ROIs original images compared to the inserted synthetic lesions ROIs.

## 5. Conclusion

A pipeline based on a previously developed and validated lesion insertion tool and FreeSurfer framework is proposed to accelerate the development, evaluation, and transition of different PET/MRI attenuation correction approaches to clinical neurological applications. Four MRI-based PET attenuation correction were evaluated against the CT attenuation map. Three types of evaluation were presented, MRAC to CTAC in lesions inserted with the background, without considering the background, and lesions ROIs in the original PET images. MRAC to CTAC in inserted lesions, with and without background, is consistent with MRAC to CTAC bias in the original reconstructed PET images. This led us to conclude that the background activity does not show an apparent effect on MRAC to CTAC bias behavior. Thus, the lesion insertion tool can be used to evaluate new MRI-based PET attenuation correction approaches without needing the measured patient PET emission data. The lesion insertion tool will be available for online users and can be used for multiple purposes.
